# Safety assessment of the microalgae *Nannochloropsis oculata*

**DOI:** 10.1016/j.toxrep.2015.03.008

**Published:** 2015-04-08

**Authors:** Michael L. Kagan, Ray A. Matulka

**Affiliations:** a Qualitas Health Ltd., Jerusalem, Israel; b Burdock Group, Orlando, FL, USA

**Keywords:** *Nannochloropsis oculata*, Omega-3, Eicosapentaenoic acid, Algae, EPA, Rat

## Abstract

*Nannochloropsis oculata* is a marine-water microalgae that is considered to be a good source of omega-3 fatty acids, specifically eicosapentaenoic acid (EPA), utilized in the production of an omega-3 oil for use as a dietary supplement. This study investigates the safety of *N. oculata* in male and female Sprague-Dawley rats administered a 0 or 10 mL/kg bw/rat *N. oculata* (10E8 viable cells/mL) suspension by oral gavage once daily for 14 consecutive days. No mortalities occurred and no signs of toxicity were observed during the study. No treatment-related effects were seen for body weight, food consumption, urinalysis, clinical chemistry, hematology, gross pathology, organ weights, or histopathology. Although statistically significant effects were noted for some endpoints, none were considered to be of toxicological significance. The *N. oculata* suspension was concluded to have no toxicity in rats, confirming that the algal strain used in the production of omega-3 oil is not pathogenic when administered orally to rats.

## 1 Introduction

*Nannochloropsis oculata* is a marine-water single-celled algae of the Eustigmatophyceae class. It is one of six species of algae found in the genus *Nannochloropsis* and was originally isolated off the coast of Scotland [1]. *Nannochloropsis* sp. has been utilized as a food source in aquaculture, providing a source of omega-3 fatty acids [2]. Recently a *N. oculata*-derived oil has been determined safe for use in dietary supplements [3]. Furthermore, the nutritional evaluation of a *Nannochloropsis* sp. found it to have high levels of protein, polyunsaturated fatty acids, and antioxidant pigments [4]. The algae is described as a phototrophic unicellular, non-zoospore producing, free-floating algae having a diameter of 2–4 μm, growing non-axenically in a temperature of 11–16 °C. The cells contain yellow-green parietal chloroplasts [1], [2].

The 14-day toxicity study was performed under Good Laboratory Practice (GLP) conditions according to the OECD [5] guidelines for the testing of chemicals. The suspension containing *N. oculata* was tested orally in rats to assess its toxicity and/or pathogenicity according to a modified study of the guidelines of OPPTS 885.3050.

## 2 Materials and methods

### 2.1 Test item

*N. oculata* suspension (Lot No. 91020140001; Qualitas Health Inc.) is a green-colored suspension of microalgae. The source biomass, *N. oculata*, was grown in shallow, open-air, plastic-lined ponds in a proprietary growth medium consisting of food grade plant fertilizers and nutrients [4]. The algae was harvested and suspended in deionized water resulting in a live algal biomass (w/w%) of <0.2% in deionized water. Toxin analyses conducted on *N. oculata* indicated that no natural product toxins were detected above detection limits including microcystins/nodularin, anatoxin-a, cylindrospermopsin, paralytic shellfish toxin/saxitoxins, okadaic acid and brevetoxins & domoic acid [6]. The *N. oculata* suspension was prepared by Qualitas Health Inc. and supplied to the lab for testing as viable cultures. The suspension was stored by refrigeration (2–8 °C).

### 2.2 Animals and housing

A total of 40 (20 male and 20 female) 7–8 week old Sprague-Dawley rats bred in-house by Advinus Therapeutics Ltd. (Bangalore, India) were housed in standard polysulfone cages (2/cage) with stainless steel top grills. Steam sterilized corn cob was used as bedding and changed along with the cage twice a week. Cages were placed on five-tier rack. The animals were acclimatized for 5 days prior to the start of treatment. Initial mean group body weights ranged from 189 to 191 g for the male rats and 153 to 155 g for the female rats. Room temperature was maintained at 20–24 °C with a relative humidity of 65–67%, a minimum of 13.7 air changes/h and a 12 h light and 12 h dark cycle. Filtered deep-bore well water and Teklad Global 14% protein rodent maintenance diet (Harlan Laboratories, An Venray, The Netherlands) were provided ad libitum. Animal handling was performed according to the requirements of the Association for Assessment and Accreditation of Laboratory Animal Care (AAALAC).

### 2.3 Experimental design

The potential toxicity or pathogenicity of a microorganism is generally assessed in accordance with the US Environmental Protection Agency Microbial Pesticide Test Guidelines OPPTS 885.3030 during which the survival and propagation of the microorganism in the rat are evaluated by culturing tissue samples, blood, and feces following a single high exposure and an adequate post-exposure observation period [7]. For the current study, the purpose was not only to determine whether or not the microorganism was toxic in its natural state, but to also determine its toxicity in the rat following ingestion. Furthermore, the current study was designed to determine if the organism could manage to replicate itself in organs and tissue of the host and eventually become a danger to the health of the animal. However, since *N. oculata* is a phototropic vegetable species and typically does not replicate without the presence of light, a modified version of the OPPTS study protocol is used that removed the steps to culture algae in tissue samples by using a 14-day oral administration of the test item to rats to provide information on its toxicity and pathogenicity.

Two groups of Sprague-Dawley rats (*n* = 10 rats/sex/group) were administered (via oral gavage) 0 and 10 mL/kg *N. oculata* suspension (providing a minimum of 10E8 viable cells/animal) respectively, once daily for 14 days. Vehicle control animals were administered by purified water. In order to determine the concentration of viable algal cells, 1 mL of the test material was evaluated daily and the algal cell counts were determined using a haemocytometer. Dead cells were identified by their loss of chlorophyll and were excluded from the cell count. There were no dead cells observed during the cell counts. The concentrations of viable algal cells in the *N. oculata* suspension were in the range of 1.10 × 10E8 to 1.32 × 10E8 viable algal cells/mL throughout the study (Table 1). Doses were administered using disposable plastic syringes, attached with a stainless steel metal feeding cannula. Following the treatment period, all animals were sacrificed on day 15.

**Table 1:** Concentration of viable algal cells during treatment.

Treatment day	Cell count (viable algal cells/mL)
1	1.21 × 10E08
2	1.28 × 10E08
3	1.32 × 10E08
4	1.25 × 10E08
5	1.27 × 10E08
6	1.14 × 10E08
7	1.18 × 10E08
8	1.23 × 10E08
9	1.12 × 10E08
10	1.15 × 10E08
11	1.10 × 10E08
12	1.16 × 10E08
13	1.20 × 10E08
14	1.16 × 10E08

Rats were observed twice daily for signs of morbidity and mortality and clinical signs until study termination. The food was analyzed and found to be below established maximum levels for heavy metals, mycotoxins (Aflatoxin B1, B2, G1 and G2), chlorinated hydrocarbons and organophosphates. The food was composed mainly of 14% protein, 4% fat and 4% fiber, as is typical for diets for this species and strain of rat. Drinking water was analyzed and found acceptable as a potable water source for the area (Bangalore, India). Body weights were recorded on day 1 prior to the test item administration and on days 4, 7, 11, and 14. Fasting body weights were recorded prior to necropsy on day 15. Food consumption was measured on days 4, 7, 11, and 14. At the end of the study, rats were fasted overnight (water allowed), anaesthetized with isoflurane, and exsanguinated.

Blood was collected from the retro-orbital sinus plexus with fine capillary tubes. An aliquot of blood was collected in tubes containing 3.2% sodium citrate solution for determination of coagulation parameters and the remaining blood was collected into k_2_EDTA and lithium heparinized tubes for hematology and clinical chemistry examinations.

Prior to sacrifice, urine was collected in urine collection tubes for all rats. Each rat was placed in specially fabricated cages overnight (water allowed) and the next morning (day 18) the urine was collected for analysis. Urinalysis parameters examined in the collected samples included color, clarity, bilirubin, glucose, ketone bodies, nitrite, proteins, pH, specific gravity, urobilinogen, and volume. Urine was also subjected to microscopic examination for sediments such as crystals, epithelial cells, erythrocytes, leukocytes and casts.

All animals were fasted overnight prior to terminal sacrifice on day 19. At the scheduled termination, all rats were euthanized by exsanguination under isoflurane anesthesia. The organs identified in Table 2 were removed, weighed and/or examined for gross pathological anomalies. Histopathological examinations were carried out on the preserved organs of both treated and control rats.

**Table 2:** Tissue collection and organ weighing.

Organ/tissue	Organ/tissue	Organ/tissue
Adrenal glands^a^	Ileum with Peyer's patches	Seminal vesicles and
All gross lesions	Jejunum	coagulating glands^a^
Aorta	Kidneys^a^	Skeletal muscle
Bone marrow smear	Liver^a^	Skin
Brain (cerebrum, cerebellum and medulla oblongata/pons)^a^	Lungs	Spinal cord (cervical, thoracic, lumbar
Cecum	Mammary gland	Spleen^a^
Colon	Mesenteric lymph nodes	Sternum with marrow
Duodenum	Esophagus	Stomach
Epididymides^a^	Ovaries^a^	Testes^a^
Esophagus	Pancreas	Thymus^a^
Eyes with optic nerve	Pituitary^a^	Thyroid with Parathyroids^a^
Femur bone with joint	Prostate^a^	Trachea
Heart^a^	Rectum	Urinary bladder
	Salivary glands	Uterus with cervix

a Weighed organs.

Data were captured using Provantis™, a toxicology data capture system. Parameters such as body weight, net bodyweight gains, food consumption, organ weights and their ratios data, clinical pathology data including hematology and clinical chemistry were analyzed using Provantis™ built-in statistical tests. All analyses and comparisons were evaluated at the 5% (*P* ≤ 0.05) level for significance. The data of both groups were subjected to pair-wise comparisons. Before performing the pair-wise comparison between the two groups, data were evaluated for normality and homogeneity. During the conduct of these analyses, when the data were found to be heterogeneous, suitable transformations were made by the inbuilt Provantis™ software and ANOVA was performed before proceeding with pair-wise tests using Student's *t*-test.

## 3 Results

**Fig. 1:**
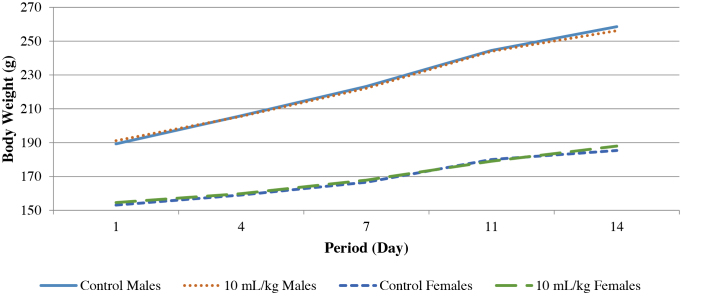
Periodical body weights of male and female rats administered *Nannochloropsis oculata* or the vehicle control by daily by oral gavage for 14 days (*n* = 10/sex/group). The data represent the mean values for each group.

No mortalities or treatment related clinical signs were observed during the treatment period. No treatment-related changes were observed in food consumption for the treated group as compared to the control group (Table 3). A statistically significant decrease in food consumption was seen in treated males during days 1–4 but returned to normal by days 4–7. The change was considered incidental and not treatment related. No treatment-related changes were observed in body weights at the tested dose in either sex during the entire treatment period (Fig. 1).

**Table 3:** Summary of food consumption (g/rat/day).

Group	Males		Females	
	0 mL/kg	10 mL/kg	0 mL/kg	10 mL/kg
Days 1–4	23.97 ± 0.46	22.73 ± 0.83*	15.29 ± 0.93	15.66 ± 0.47
Days 4–7	23.65 ± 1.10	23.59 ± 0.96	15.10 ± 0.75	16.08 ± 0.89
Days 7–11	22.90 ± 0.75	23.31 ± 1.15	16.26 ± 1.04	16.40 ± 0.65
Days 11–14	22.68 ± 0.62	22.49 ± 0.48	15.88 ± 0.91	15.85 ± 0.43

All data are presented as mean values ± standard deviations with *N* = 5.

* Statistically significant from the vehicle control group (*P* ≤ 0.05).

Data from the hematology, coagulation, and clinical chemistry analyses performed on day 15 are presented in Table 4. Statistically significant changes in hematology included increased absolute monocytes in treated males and increased neutrophils (%) and decreased white blood cells and absolute lymphocytes in treated females. These changes were considered incidental due to the minor magnitude of the changes and/or the changes being within the physiological range (historical control data for the statistically significant parameters are provided in Table 5). For coagulation parameters, an increase in prothrombin time (5%) was seen in treated females and was considered an incidental finding. No other changes in coagulation parameters were seen. Clinical chemistry values including blood urea nitrogen (BUN), total protein, inorganic phosphorous, calcium, albumin, globulin, and A/G ratio were statistically different in treated males and/or females when compared to controls. All of the changes that reached statistical changes were of a low magnitude and within the physiological range of the rat model, and therefore were considered toxicologically insignificant. No statistically significant changes were seen in any of the urine parameters analyzed in terminally sacrificed animals.

**Table 4:** Summary of hematology, coagulation, and clinical chemistry values on day 15.

Parameter	Males		Females	
	0 mL/kg	10 mL/kg	0 mL/kg	10 mL/kg
Hematology				
WBC (10^9^/L)	8.37 ± 1.27	9.22 ± 1.44	7.85 ± 1.30	6.70 ± 1.14*
RBC (10^12^/L)	8.39 ± 0.20	8.52 ± 0.29	8.31 ± 0.21	8.22 ± 0.21
HGB (g/L)	157 ± 5	156 ± 4	156 ± 5	154 ± 4
HCT (L/L)	0.526 ± 0.011	0.529 ± 0.014	0.514 ± 0.017	0.510 ± 0.015
MCV (fL)	62.7 ± 0.8	62.1 ± 0.8	61.8 ± 0.9	62.1 ± 1.2
MCH (pg)	18.7 ± 0.6	18.3 ± 0.5	18.8 ± 0.3	18.7 ± 0.2
MCHC (g/L)	298 ± 7	295 ± 5	304 ± 4	302 ± 4
Plat (10^9^/L)	1088 ± 73	1071 ± 91	1036 ± 95	1110 ± 124
Neut (%)	10.0 ± 3.2	10.8 ± 2.9	7.8 ± 1.3	9.5 ± 1.9*
Lymp (%)	86.0 ± 3.7	85.0 ± 3.0	87.5 ± 2.8	86.2 ± 2.1
Mono (%)	1.8 ± 0.5	2.0 ± 0.4	2.5 ± 2.2	2.1 ± 1.6
Eosi (%)	0.6 ± 0.6	0.6 ± 0.5	0.6 ± 0.2	0.7 ± 0.2
Baso (%)	0.5 ± 0.3	0.4 ± 0.1	0.3 ± 0.1	0.3 ± 0.1
Neut A (10^9^/L)	0.81 ± 0.16	1.01 ± 0.34	0.62 ± 0.15	0.65 ± 0.22
Lymp A (10^9^/L)	7.23 ± 1.34	7.83 ± 1.24	6.85 ± 0.95	5.77 ± 0.97*
Mono A (10^9^/L)	0.14 ± 0.03	0.18 ± 0.04*	0.22 ± 0.25	0.14 ± 0.08
Eosi A (10^9^/L)	0.05 ± 0.04	0.05 ± 0.04	0.04 ± 0.01	0.04 ± 0.01
Baso A (10^9^/L)	0.04 ± 0.03	0.03 ± 0.01	0.03 ± 0.01	0.02 ± 0.01

Coagulation				
PT (s)	16.9 ± 1.2	17.2 ± 0.6	17.7 ± 0.5	18.5 ± 0.6*
APTT (s)	14.2 ± 2.2	13.7 ± 2.8	12.2 ± 1.5	11.6 ± 2.9

Clinical chemistry				
Glu (mmol/L)	7.44 ± 0.73	7.16 ± 0.60	6.87 ± 0.54	7.12 ± 0.32
BUN (mmol/L)	3.94 ± 0.36	4.58 ± 0.94*	5.92 ± 0.81	5.11 ± 0.65*
Creat (μmol/L)	24 ± 9	21 ± 4	25 ± 8	21 ± 6
AST (U/L)	93 ± 17	92 ± 11	94 ± 9	94 ± 6
ALT (U/L)	61 ± 11	55 ± 6	49 ± 6	50 ± 4
GGT (U/L)	0 ± 1	1 ± 1	2 ± 1	1 ± 1
ALP (U/L)	177 ± 10	180 ± 16	117 ± 17	120 ± 15
T. Bil (μmol/L)	1.70 ± 0.78^a^	1.50 ± 0.54	1.25 ± 0.58^b^	0.91 ± 0.28^b^
T. Chol (mmol/L)	3.34 ± 0.15	3.41 ± 0.27	3.49 ± 0.17	3.49 ± 0.20
Trig (mmol/L)	0.74 ± 0.11	0.71 ± 0.08	0.66 ± 0.16	0.59 ± 0.06
T. Pro (gL)	64.1 ± 1.8	69.4 ± 4.8*	70.8 ± 1.6	66.9 ± 1.2*
Alb (g/L)	41.0 ± 1.3	40.4 ± 1.2	40.2 ± 2.8	41.7 ± 0.8*
Pi (mmol/L)	2.95 ± 0.11	2.78 ± 0.19*	2.44 ± 0.17	2.37 ± 0.10
Ca (mmol/L)	2.47 ± 0.11	2.32 ± 0.11*	2.43 ± 0.21	2.48 ± 0.10
Na (mEq/L)	149.4 ± 1.7	150.5 ± 0.7	148.4 ± 1.5	149.3 ± 1.8
K (mEq/L)	3.90 ± 0.21	3.86 ± 0.24	3.47 ± 0.26	3.47 ± 0.21

WBC, white blood corpuscles; RBC, red blood corpuscles; HGB, haematoglobin; HCT, haematocrit; MCV, mean corpuscular volume; MCH, mean corpuscular hemoglobin; MCHC, mean corpuscular hemoglobin concentration; Plat, platelets; Neut, neutrophils; Lymp, lymphocytes; Mono, monocytes; Eosi, eosinophils; Baso, basophils; PT, prothrombin time; APTT, activated partial thromboplastin time; Glu, glucose; BUN, blood urea nitrogen; Creat, creatine; AST, aspartate aminotransferase; ALT, alanine aminotransferase; GGT, gamma glutamyl transpeptidase; ALP, alkaline phosphatase; T. Bil, total bilirubin; T. Chol, total cholesterol; Trig, triglycerides; T. Pro, total plasma protein; Alb, albumin; Pi, inorganic phosphorous; Ca, calcium; Na, sodium; K, potassium.

Data are presented as mean values ± standard deviations with *N* = 10.

* Statistically significant from the vehicle control group (*P* ≤ 0.05).

a *N* = 8 (two values below the lower limit of Quantification for T. Bil (0.40 μmol/L) were not considered for analysis).

b *N* = 9 (one value below the lower limit of Quantification for T. Bil (0.40 μmol/L) was not considered for analysis).

**Table 5:** Historical control data for statistically significant hematology/clinical chemistry parameters.

Parameter	Historical control values (mean values)	Historical control (ranges)
Hematology and coagulation		
Monocyte absolute – males (G/L)	0.63	0.04–6.60
White blood cells (WBC) – females (G/L)	8.44	3.76–13.87
Neutrophil % – females (%)	8.36	0.67–17.60
Lymphocyte absolute – females (G/L)	13.18	2.91–83.20
Prothrombin time – females (s)	16.50	13.20–19.20

Clinical chemistry		
Blood urea nitrogen – males (mmol/L)	4.81	1.97–8.04
Total protein – males (g/L)	61.93	47.50–72.90
Inorganic phosphorous – males (mmol/L)	2.86	2.25–3.70
Calcium – males (mmol/L)	16.07	2.32–154.30
Blood urea nitrogen – females (mmol/L)	5.62	3.11–9.18
Total protein – females (g/L)	64.51	48.00–79.00
Albumin – females (g/L)	39.56	9.60–60.70

Organ weights		
Kidneys – absolute wt – females (g)	1.33	0.74–1.97

When compared to the control groups, no significant changes attributed to treatment were seen in organ weights or organ to body weight ratios for treated males or females (Table 6, Table 7). A marginal but statistically significant increase in absolute kidney weights was seen in treated females (7%). This increase was not statistically significant in relative kidney weights. In addition, no correlating changes in clinical observations or histopathological changes were seen. Therefore, the kidney weight change was considered to be incidental.

**Table 6:** Summary of terminal fasting body weights and absolute organ weights on day 15.

Group	Males		Females	
	0 mL/kg	10 mL/kg	0 mL/kg	10 mL/kg
Terminal fasting BW (g)	242.47 ± 7.85	241.55 ± 8.90	175.73 ± 11.93	179.52 ± 8.19
Adrenals (g)	0.0494 ± 0.0057	0.0504 ± 0.0050	0.0635 ± 0.0039	0.0635 ± 0.0063
Brain (g)	1.7788 ± 0.0721	1.7615 ± 0.0479	1.6966 ± 0.0495	1.6655 ± 0.0635
Epididymides (g)	0.7626 ± 0.0656	0.7815 ± 0.0693	–	–
Heart (g)	0.9614 ± 0.0661	0.9400 ± 0.0542	0.7365 ± 0.0728	0.7516 ± 0.0689
Kidneys (g)	1.7076 ± 0.1248	1.7031 ± 0.1289	1.1664 ± 0.0970	1.2517 ± 0.0825*
Liver (g)	7.8023 ± 0.6408	7.9786 ± 0.3449	5.7662 ± 0.6303	5.8339 ± 0.4218
Ovaries	–	–	0.0829 ± 0.0151	0.0903 ± 0.0123
Pituitary (g)	0.0106 ± 0.0011	0.0102 ± 0.0009	0.0119 ± 0.0016	0.0123 ± 0.0009
Prostate (g)	0.6334 ± 0.1241	0.6051 ± 0.0892	–	–
Seminal vesicles & coagulating glands (g)	0.9122 ± 0.1593	0.9074 ± 0.1118	–	–
Spleen (g)	0.6686 ± 0.0491	0.6749 ± 0.0606	0.5584 ± 0.0940	0.5802 ± 0.0760
Testes (g)	3.2168 ± 0.1936	3.2735 ± 0.2095	–	–
Thymus (g)	0.5265 ± 0.0494	0.5356 ± 0.0921	0.4463 ± 0.1134	0.4184 ± 0.1130
Thyroid with parathyroids (g)	0.0287 ± 0.0060	0.0299 ± 0.0049	0.0268 ± 0.0060	0.0249 ± 0.0029
Uterus with cervix (g)	–	–	0.4234 ± 0.0523	0.5206 ± 0.2208

All data are presented as mean values ± standard deviations with *N* = 10.

* Statistically significant from the vehicle control group (*P* ≤ 0.05).

**Table 7:** Summary of organ to body weight ratios on day 15.

Group	Males		Females	
	0 mL/kg	10 mL/kg	0 mL/kg	10 mL/kg
Adrenals (%)	0.0204 ± 0.0027	0.0209 ± 0.0025	0.0363 ± 0.0033	0.0354 ± 0.0037
Brain (%)	0.7337 ± 0.0219	0.7300 ± 0.0289	0.9704 ± 0.0846	0.9301 ± 0.0660
Epididymides (%)	0.3151 ± 0.0320	0.3235 ± 0.0242	–	–
Heart (%)	0.3968 ± 0.0287	0.3892 ± 0.0177	0.4186 ± 0.0208	0.4181 ± 0.0249
Kidneys (%)	0.7040 ± 0.0405	0.7053 ± 0.0495	0.6646 ± 0.0469	0.6985 ± 0.0520
Liver (%)	3.2141 ± 0.1737	3.3056 ± 0.1533	3.2772 ± 0.2061	3.2481 ± 0.1504
Ovaries (%)	–	–	0.0473 ± 0.0091	0.0504 ± 0.0075
Pituitary (%)	0.0044 ± 0.0005	0.0042 ± 0.0004	0.0068 ± 0.0009	0.0069 ± 0.0006
Prostate (%)	0.2612 ± 0.0498	0.2500 ± 0.0315	–	–
Seminal vesicles & coagulating glands (%)	0.3770 ± 0.0698	0.3761 ± 0.0481	–	–
Spleen (%)	0.2757 ± 0.0180	0.2794 ± 0.0236	0.3161 ± 0.0350	0.3227 ± 0.0340
Testes (%)	1.3264 ± 0.0606	1.3562 ± 0.0903	–	–
Thymus (%)	0.2169 ± 0.0163	0.2216 ± 0.0370	0.2521 ± 0.0500	0.2311 ±0.0524
Thyroid with parathyroids (%)	0.0118 ± 0.0025	0.0124 ± 0.0019	0.0152 ± 0.0031	0.0139 ± 0.0017
Uterus with cervix (%)	–	–	0.2419 ±0.0323	0.2909 ± 0.1259

All data are presented as mean values ± standard deviations with *N* = 10.

* Statistically significant from the vehicle control group (*P* ≤ 0.05).

Compared to their respective control groups, no test item-related gross pathological lesions were evident in male or female rats at terminal necropsy (day 15). No incidental findings were seen in females. In treated males a single incidence of unilateral testis focal reddish discoloration was seen. No correlating changes were seen microscopically. The discoloration was determined to be incidental. No other incidental findings were seen in males. Microscopic findings (accessory cortical tissue in adrenal glands, esophageal inflammatory foci, cardiac inflammatory focus, renal basophilic or cystic tubules, hepatocellular inflammatory focus, pulmonary inflammatory focus, pancreatic acinar cell apoptosis, prostate lymphocytic infiltration, spinal cord keratin cyst, stomach dilated gland glandular mucosa, thymus hemorrhage, inflammation or epithelial cyst with eosinophilic crystals, thyroid gland ectopic thymus, and uterine dilation) occurred at low frequencies in the control and treated animals and were not considered treatment related (Table 8).

**Table 8:** Summary of histopathological findings for male and female rats following exposure to *Nannochloropsis oculata* for 14 days.

Group	Males		Females	
	0 mL/kg	10 mL/kg	0 mL/kg	10 mL/kg
Number of animals per group	10	10	10	10
Adrenal glands				
Accessory cortical tissue	0	0	0	1
Esophagus				
Inflammatory focus; muscularis (minimal)	0	1	1	1
Heart				
Inflammatory focus (minimal)	0	1	0	0
Kidneys				
Basophilic tubules (minimal)	2	1	0	0
Cystic tubule – solitary	0	0	1	0
Liver				
Inflammatory focus (minimal)	0	1	0	0
Lungs				
Inflammatory focus (minimal)	0	0	0	1
Pancreas				
Increased acinar cell apoptosis (minimal)	2	1	0	0
Pituitary gland				
Cyst; pars distalis	0	0	1	0
Prostate				
Lymphocytic infiltration (minimal)	2	1	–	–
Rectum				
Nematode	1	0	0	0
Spinal cord				
Keratin cyst	0	0	0	1
Stomach				
Dilated gland – solitory; glandular mucosa	0	0	0	1
Thymus				
Hemorrhage (minimal)	0	0	0	1
Inflammation (mild)	0	0	0	1
Epithelial cysts with eosinophilic crystals	1	1	2	1
Thyroid gland				
Ectopic thymus	0	2	1	0
Uterus with cervix				
Dilation (mild)	–	–	0	2

All data are presented as number of animals affected.

Under the conditions of this study, oral gavage administration of viable *N. oculata* suspension at the dose volume of 10 mL/kg (providing at least 1 × 10E8 viable cells) to male and female rats for 14 days did not cause any treatment-related effects.

## 4 Discussion

Prior to this study, the toxicity of *Nannochloropsis* species algae has been assessed in rats following acute and subchronic (up to 60 days) administration [8], [9], [10] as well as in pregnant rats during mating and through pregnancy and lactation [11]. Acute toxicity tests in rats administered biomass of *N. oculata* by oral gavage revealed no effects (LD_50_ ≥ 12 g/kg) [8], [9]. Similarly, no treatment-related effects were seen in rats treated with 3000 or 6000 mg/kg/day *N. oculata* biomass by oral gavage daily for 60 days (NOAEL ≥ 6000 mg/kg/day) [8]. However, this previously conducted study only focused on the potential nephrotoxicity or hepatotoxicity of a nonviable *N. oculata* biomass, not to the potential pathogenicity of the viable organism. No treatment-related effects were seen in rats fed 10,000 mg/kg/day whole freeze-dried *Nannochloropsis* (species not stated), algal lipid extract (3500 mg/kg/day) or algal residue (6500 mg/kg/day) daily for 30 days [10]. However, Nuno et al., [9] reported weight loss in hyperglycemic male rats treated with a freeze-dried culture of *N. oculata* by oral gavage (250 mg/kg/day) daily for 8 weeks. Examination of intestinal tissue sections revealed the presence of intestinal atrophy and gastrointestinal damage. The authors noted that the adverse effects may have been due to the algae's cellular structure, as the cell wall of *N. oculata* is rigid and relatively thick, and when freeze-dried could adversely impact the epithelium, lactic acid bacteria counts, and nutrient absorption [9]. No reproductive or developmental effects were seen following the treatment of pregnant and lactating rats with 2000 mg/kg/day *Nannochloropsis* algae in the diet [11].

Here we describe the nonclinical toxicity of viable *N. oculata* in rats following daily administration in rats by oral gavage for 14 days. No treatment-related effects were seen in male or female rats following daily oral treatment with 10 mL/kg, providing at least 1 × 10E8 viable algal cells to each animal. A transient decrease in food consumption occurred at days 1–4 in the male rats, but returned to the control range for the remainder of the study. Non-viable *N. oculata* biomass has been provided to Sprague-Dawley rats for 60 days with significant increases in body weight gains, compared to control rats [8]. However, it was not stated that the diets in this study were isocaloric. Statistically significant changes seen in the hematological parameters were not indicative of a response to infection, as the changes were both increased (i.e., absolute monocytes in the males and increased neutrophils in the females) and decreased (i.e., white blood cells and absolute lymphocytes), and were minor in magnitude. In the same manner, the clinical chemistry value changes that reached significance were not consistent between the male and female groups, were within the physiological range of the Sprague-Dawley rat for the laboratory, and did not correlate with any histological changes in the organs. This is the first known study which evaluated the potential toxicity or pathogenicity of the microalgae *N. oculata*. Other microalgae are known to produce algal toxins that can cause animal and human toxicity. Administration of the viable *N. oculata* cells shows that this species of microalgae does not produce toxins that hinder the growth of this rat model when consumed over a 14-day period.

Because it is a rich source of eicosapentaeneoic acid (EPA), the microalgae *N. oculata* is being utilized in the production of Almega PL™ (Qualitas Health, Ltd.), an EPA-rich omega-3 oil isolated for use as a dietary supplement ingredient. In addition to the 14-day study on *N oculata* presented here, a battery of nonclinical studies indicating the safety of the algal oil at levels up to 2000 mg/kg/day has recently been published [12]. As shown in several published preclinical studies, consumption of *N. oculata* biomass for up to 60 days did not cause any adverse effects. However, one study did indicate that gavage administration of freeze-dried *N. oculata* biomass caused gastrointestinal damage most likely due to the rigid cell wall of *N. oculata*. Because EPA supplements derived from *N oculata* would consist of oil extracted from the algae, and not the algae itself, the rigid cell wall would not be a safety issue.

In conclusion, treatment with viable *N. oculata* suspension at a dose volume of 10 mL/kg (providing at least 1 × 10E8 viable algal cells/animal) to male and female rats for 14 days did not cause any treatment-related effects. Based on the available data, *N. oculata* used in the development of an EPA-delivering dietary supplement is not toxigenic or pathogenic when administered orally to rats.

## Conflict of interest

Michael L. Kagan is an employee of Qualitas Health Ltd. Ray A. Matulka declares no potential conflicts of interest with respect to the research, authorship, and/or publication of this article.

## Transparency document

Transparency document
